# Mosunetuzumab and lymphoma: latest updates from 2022 ASH annual meeting

**DOI:** 10.1186/s13045-023-01462-0

**Published:** 2023-06-28

**Authors:** Yang Cao, Emanuela C. Marcucci, Lihua E. Budde

**Affiliations:** 1 Department of Hematology, Tongji Hospital, Tongji Medical College, Huazhong University of Science and Technology, Wuhan, Hubei China; 2 Department of Hematology & Hematopoietic Stem Cell Transplantation, City of Hope National Medical Center, Duarte, CA 91010 USA

## Abstract

Bispecific antibodies are emerging as a promising new immunotherapy modality and are actively being evaluated in clinical trials for patients with lymphoma. As the first BsAb to receive regulatory approval for lymphoma, mosunetuzumab, an antiCD20/anti-CD3 BsAb, is an exciting new option for patients with relapsed or refractory (R/R) follicular lymphoma. The approval was based on results from an international, multicenter, phase 2 trial in patients with relapsed or refractory (R/R) follicular lymphoma following at least 2 prior lines of systemic therapy. Mosunetuzumab demonstrated remarkable efficacy with an overall response rate of 80% and complete response rate of 60%. Here we provided an overview of the latest clinical data on mosunetuzumab in lymphoma presented at the 2022 ASH Annual Meeting.

**To the Editor,**

Bispecific antibodies (BsAbs) are emerging as a promising new immunotherapy modality and are actively being evaluated in clinical trials for patients with lymphoma. As the first BsAb to receive regulatory approval for lymphoma, mosunetuzumab, an antiCD20/anti-CD3 BsAb, is an exciting new option for patients with relapsed or refractory (R/R) follicular lymphoma (FL). The approval was based on results from an international, multicenter, phase 2 trial in patients with relapsed or refractory (R/R) FL following at least 2 prior lines of systemic therapy [1, 2]. Mosunetuzumab demonstrated remarkable efficacy with an overall response rate (ORR) of 80% and complete response (CR) rate of 60%. Here we provided an overview of the latest clinical data on mosunetuzumab in lymphoma presented at the 2022 ASH Annual Meeting (ASH2022).

Durable response in patients receiving mosunetuzumab.

Bartlett et al. [1] reported update results of mosunetuzumab from the pivotal phase 2 study after a median 28.3 months of follow-up. The 24-month progression free survival (PFS), overall survival (OS) and duration of CR were 48% (95% CI 36–60), 87% (95% CI 80–94), 63% (95% CI 38–88) respectively. Whole exome sequencing analysis of 51 available baseline lymphoma samples showed that clinically meaningful response rates were observed in patients with common mutations including those associated with poor prognosis such as TP53, KMT2D, EZH2, and BCL-2. No new serious adverse events (AEs), Grade ≥ 3 AEs, or treatment-related AEs were reported with the additional 10 months more follow up than the original report [2]. Cytokine release syndrome (CRS) of all grades was seen in 44% with 26% grade 1 and 17% grade 2. No correlation was observed between the occurrence of CRS and tumor response. Mosunetuzumab related AEs leading to treatment discontinuation was seen in 2% of patients.

McGough SF et al. [3]. conducted an external control cohort study based on real world data from US patients with r/r FL who received third-line or later treatments and met the eligibility criteria of the pivotal phase 2 trial leading to mosunetuzumab approval. There was a significant treatment benefit associated with mosunetuzumab for CR rate (odds ratio [OR], 3.18; 95% confidence interval [CI] 1.41—7.17) and OS (hazard ratio [HR], 0.43; 95% CI 0.19–0.94), supporting mosunetuzumab use in this clinical setting.

Subcutaneous administration of mosunetuzumab.

To reduce CRS incidence and improve the convenience of mosunetuzumab, subcutaneous administration as an alternative to the current I.V. route is under clinical testing. Budde et al. [4] presented updated safety and efficacy data from an ongoing study testing subcutaneous (SC) administration in patients (N = 87) with B non-Hodgkins lymphoma (NHL) with a median follow up of 10.2 months. Fixed duration SC mosunetuzumab given at various dose levels demonstrated good safety profile with no dose limiting toxicity observed. Six of 11 (54.5%) patients with indolent NHL and 15/76 (19.7%) patients with aggressive NHL achieved a CR. Based on exposure–response considerations regarding tolerability and clinical activity, 5/45/45 mg was chosen as the recommended phase 2 dose. A phase 2 expansion cohort in patients with follicular lymphoma is currently enrolling patients.

Mosunetuzumab in elderly patients.

Olszewski et al. [5] report updated efficacy and safety outcomes with at least 1 year of follow-up after the end of treatment (EOT) from a Phase I/II, multicenter study (NCT03677154) evaluating mosunetuzumab monotherapy in elderly/unfit patients with previously untreated DLBCL. Fifty-four patients with a median age of 83 years (range: 65–100) and median follow-up of 23.3 months were treated and evaluable. Best ORR and CR rates were 56% (30/54) and 43% (23/54), respectively. The 12-month PFS rate was 39% (95% CI 25.8–52.8). The treatment was well tolerated with only one patient discontinued from the study due to non mosunetuzumab related AEs. None developed immune effector cell-associated neurotoxicity (ICANS). All CRS events were low grade (26%). Exploratory biomarker analyses of tumor composition revealed increased levels of CD8 + T cells at baseline in responders compared to non-responders. Results from this study pave the way to test mosunetuzumab in combination with CHOP or other frontline multi-agent regimens (Tables 1, 2).

**Table 1:** Outcomes of clinical trials using mosunetuzumab in indolent lymphomas

Disease	Regimen	Dose (mg)	ORR/CR	PFS/OS/mDOR (95% CI)	CRS/ICANS	Reference
FL, ≥ 3 line	M	I.V. 8–17 cycles 1/2/60/30	80%/60%	24-month PFS, 48% (36–60) 24-month OS, 87% (80–94) mDOR, not reached	CRS: 44%, 2% ≥ grade 3 ICANS: 4.4% (no ≥ grade 3)	1, 2
iNHL > 1 line	M	Subq 8–17 cycles G1: 5/15/45 G2: 5/45/45 G3: 5/90/45	82%/55%	mDOR, not reached	*CRS: 28%, 0% ≥ grade 3 *ICANS: 3.4% (3 grade 1)	4
FL	M + len	M: I.V. 12 cycles Len: 11 cycles (C2-12)	90%/72%	n/a	CRS: 28%, 0% ≥ grade 3 ICANS: n/a	9, 10

^*^All patients enrolled in group 1 and 2

**Table 2:** Outcomes of clinical trials using mosunetuzumab in aggressive lymphomas

Disease	Regimen	Dosing (mg)	ORR/OR	PFS/OS/DOR	CRS/ICANS	Reference
aLBCL 1st line	M	M I.V. 8–17 cycles 1/2/13.5 1/2/30	56%/43%	12 months PFS 39% (25.8–52.8) 12 months OS 75% (63.4–86.8)	CRS: 44%, 2% ≥ grade 3 ICANS: 4.4%, 0% ≥ grade 3	5
aNHL	M	Subq. 8–17 cycles 5/15/45 5/45/45 5/90/45	37%/20%	Median DOR 6.1 months (4.6-NE)	CRS: 28%, 0% ≥ grade 3 ICANS: 3.4%, 0% ≥ grade 3	4
aLBCL > 1 line	M + Pola	M: I.V. 8–17 cycles Pola: 6 cycles	65%/48%	Median PFS: 8.9 months (95% CI: 3.5, NE)	CRS: 18%, 0% ≥ grade 3 ICANS: 7.9%, 3.2% ≥ grade 3	6

M: mosunetuzumab; FL: follicular lymphoma; B-NHL: B non-Hodgkin’s lymphoma; DLBCL: diffuse large B cell lymphoma; iNHL: indolent non-Hodgkin’s lymphoma; aNHL: aggressive non-Hodgkin’s lymphoma; aLBCL: aggressive large B cell lymphoma; ORR: overall response rate; CR: complete response; PFS: progression free survival; OS: overall survival; Len: lenalidomide

CRS: cytokine release syndrome; ICANS: Immune effector cell-associated neurotoxicity syndrome;

Olszewski et al. [6] presented a subgroup analysis of the efficacy and safety of mosunetuzumab (M) in combination with polatuzumab (Pola), an anti-CD79b antibody drug conjugate, in patients aged < 65 and ≥ 65 years with R/R aggressive B cell lymphoma (aBCL) from the Phase Ib dose-escalation and Phase II dose-expansion cohorts of the GO40516 study. With a median follow-up of 5.3 months (range: 0.7–23.7 months), 60 pts had received M-Pola: 24 (40%) were aged < 65 years and 36 (60%) were aged ≥ 65 years. Compared with younger patients, those aged ≥ 65 years had a numerically higher ORR (72% [95% CI 55–86] vs. 54% [95% CI 33–75]) and CR rate (56% [95% CI 38–72] vs. 38% [95% CI 19–59]).CRS were all low grade with no grade 3 and above and comparable in pts aged ≥ 65 and < 65 years (17% vs. 21%). Comparable rates of CRS and serious AEs were observed across age groups. This report demonstrated that the M + Pola regimen is effective with a manageable safety profile in older patients. Enrollment in the Phase II study with no mandatory hospitalization is ongoing. Additionally, a phase III trial evaluating M-Pola combination versus Rituximab in combination with gemcitabine plus oxaliplatin in patients with R/R aggressive aBCL is underway [7].

Biomarkers and response to Mosunetuzumab in combination with lenalidomide.

Lenalidomide is commonly utilized in combination with other agents for patients with FL due to its notable immune modulatory effect. Olszewski et al. [8] reported a trial in progress of combining mosunetuzumab with lenalidomide in front line use for FL and marginal zone lymphoma. Morschhauser et al. [9] reported CR rate of 77% previously in a phase 1b study of evaluating mosunenetuzumab and lenalidomide combination in patients with R/R FL. Bishton et al. [10] examined the baseline biomarkers in 29 patients treated in this study. In line with the mechanism of action of both drugs, this combination led to increased percentage of T cells and NK cells expressing activation (CD69, granzyme B) and maturation markers (i.e. HLA-DR). The addition of lenalidomide starting from cycle 2 did not increase the level of IL-6. This correlates with the low incidence of clinical CRS as previously reported and is consistent with the notion that CRS predominantly occurs in cycle 1 in studies using single agent mosunetuzumab. Among the 4 patients with disease progression on treatment, three of them had a loss of CD20 with one of them having CD20 negative disease prior to treatment. This result confirms that CD20 loss is a part of mechanism of resistance and CD20 negative lymphoma defined as < 5% CD20 + PAX5 + lymphoma cells, does not benefit from mosunetuzumab treatment [11].

In conclusion, mosunetuzumab represents a new form of immunotherapy for treating lymphoma. Promisng interim results from various clinical trials have demonstrated its effective anti-lymphoma properties and manageable safety profile. These findings support the need for further investigation into use of mosunetuzumab monotherapy in earlier lines of treatment and in combination with other agents like lenalidomide and polatuzumab. To ensure the appropriate and optimal untilization of mosunetuzumab in the clinical setting, it is essential to identify prognostic factors that are associated with durable response to mosunetuzumab treatment. Additionally, dedicated research efforts aimed at understanding the complex mechanisms of resistance in patients receiving mosunetuzumab will guide the development of next generation of bsAb therapies, and the evaluation of innovative combination treatment strategies in lymphoma patients.

## Data Availability

The material supporting the conclusion of this study has been included within the article.

## Abbreviations

ASH: American Society of Hematology
ALL: Acute lymphoblastic leukemia
CAR: Chimeric antigen receptor
CRS: Cytokine release syndrome
ICANS: Immune effector cell-associated neurotoxicity syndrome
CR: Complete response
GvHD: Graft vs host disease
ORR: Overall response rate
PFS: Progression free survival
OS: Overall survival

## References

[1] Bartlett NL, Sehn LH, Matasar MJ, Schuster SJ, Assouline SE, Giri P (2022). Mosunetuzumab monotherapy demonstrates durable efficacy with a manageable safety profile in patients with relapsed/refractory follicular lymphoma who received ≥2 prior therapies: updated results from a pivotal phase II study. Blood.

[2] Budde LE, Sehn LH, Matasar M, Schuster SJ, Assouline SE, Giri P (2022). Safety and efficacy of mosunetuzumab, a bispecific antibody, in patients with relapsed or refractory follicular lymphoma: a single-arm, multicentre, phase 2 study. Lancet Oncol.

[3] McGough SF, Shamas N, Wang J, Jaber M, Swarup B, Zumofen MB (2022). An external control for mosunetuzumab using real-world data in follicular lymphoma in the third or subsequent lines of systemic therapy. Blood.

[4] Budde LE, Bartlett NL, Giri P, Schuster SJ, Assouline SE, Yoon S (2022). Subcutaneous Mosunetuzumab is active with a manageable safety profile in patients (pts) with relapsed/refractory (R/R) B-cell non-hodgkin lymphomas (B-NHLs): updated results from a phase I/II study. Blood.

[5] Olszewski AJ, Avigdor A, Babu S, Levi I, Eradat H, Abadi U (2022). Mosunetuzumab monotherapy continues to demonstrate promising efficacy and durable complete responses in elderly/unfit patients with previously untreated diffuse large B-cell lymphoma. Blood.

[6] Olszewski AJ, Budde LE, Chavez J, Ghosh N, Kamdar M, Lossos IS (2022). Mosunetuzumab with polatuzumab vedotin is effective and has a manageable safety profile in patients aged <65 and ≥65 years with relapsed/refractory diffuse large B-cell lymphoma (R/R DLBCL) and ≥1 prior therapy: subgroup analysis of a phase Ib/II study. Blood.

[7] Westin J, Olszewski AJ, Fogliatto LM, Kim W, Shin H, Wu H (2022). SUNMO: a phase III trial evaluating the efficacy and safety of mosunetuzumab in combination with polatuzumab vedotin versus rituximab in combination with gemcitabine plus oxaliplatin in patients with relapsed or refractory aggressive B-cell non-hodgkin lymphoma. Blood.

[8] Olszewski AJ, Huntington SF, Bannerji R, Ollila TA, McMahon J, Dubielecka PM (2022). Mosunetuzumab with lenalidomide augmentation as first-line therapy for follicular (FL) and marginal zone lymphoma (MZL). Blood.

[9] Morschhauser F, Bishton M, Eyre TA, Bachy E, Cartron G, Ysebaert L (2021). Mosunetuzumab in combination with lenalidomide has a manageable safety profile and encouraging activity in patients with relapsed/refractory follicular lymphoma: initial results from a phase Ib study. Blood.

[10] Bishton M, Woestmann C, Morschhauser F, Eyre TA, Shin M, Schroeder A (2022). Analysis of immune pharmacodynamic and baseline biomarkers in patients with relapsed or refractory follicular lymphoma treated with mosunetuzumab in combination with lenalidomide. Blood.

[11] Schuster SJ, Huw L, Bolen CR, Assouline SE, Bartlett NL, Budde LE (2022). Characterization of CD20 expression loss as a mechanism of resistance to mosunetuzumab in patients with relapsed/refractory B-cell non-Hodgkin lymphomas. J Clinic Oncol.

